# The American Social Hygiene Association

**Published:** 1915-05

**Authors:** 


### The American Social Hygiene Association.

The American Social Hygiene Association has been offered a prize of $1000 by the Metropolitan Life Insurance Company to be awarded to the author of the best original pamphlet on social hygiene for adolescents between the ages of twelve and sixteen years, approved by a committee of judges to be selected by the Association.

The work of this Association is directed to the promotion and guidance of sex education, the establishment of a single standard of morals, and the suppression of prostitution and its attendant evils—venereal disease, mental and moral degeneracy, and economic waste. The Association believes that the problems involved cannot be successfully attacked without thorough knowledge of their causes, extent, and relation to other social problems. It seeks to be guided by reason and the facts, and desires the co-operation of all those who strive for social and moral progress.

Competition for this prize is open to all.

The Metropolitan Life Insurance Company desires to use the winning pamphlet among its industrial policyholders.

The Committee of Judges will conduct the competition in accordance with the following conditions:

Contest closes July 31, 1915, at midnight; any manuscript received later will not be considered.

Manuscripts should not exceed 3500 words and must be in English and must not have been previously published.

Manuscripts must be typewritten on one side only of plain white paper 8 inches by 10½ inches.

Manuscripts must be paragraphed and punctuated for submission as "copy" to printer.

Each manuscript must bear some identifying mark or pen-name, but not the name of the author.

The author's name and address, and the identifying mark or pen-name should be in a sealed envelope, accompanying the manuscript; the face of the envelope should bear the mark or pen-name only.

More than one manuscript may be submitted by the same author.

The winning manuscript, in consideration of the award of $1000, becomes the property of the donor of the prize, all rights therein being surrendered by the author.

The right to purchase any manuscript submitted, at the rate of 5 cents a word, is reserved by the Metropolitan Insurance Company and by the American Social Hygiene Association.

Any manuscript not winning the prize or purchased will be returned to the author if return postage is provided.

Address manuscripts and requests for further information to The American Social Hygiene Association, 105 West Fortieth Street, New York City.

